# Supplementary material for: Effects of ocean acidification on the dissolution rates of reef-coral skeletons
Source: PeerJ. 2013 Nov 21;1:e208. doi: 10.7717/peerj.208 (PMC3840418; doi:10.7717/peerj.208)
Supplement: Appendix S1 — Matlab code for Eq. (1). [file peerj-01-208-s001.docx]

**Appendix**

function dA = accrete(t, A)

global a bS cD; dA = [(a*A)/A + bS- (cD*A)/A];

% For perforate skeletons

hold on; global a bS cD; timespan= [1990 2100];

% at 4 kg CACO3 m2 year is 3 mm

a= .3; bS= .1; cD= .45;

y0=-1;fname= 'accrete';[t A] = ode45(fname, timespan, y0);hold on

plot(t, A,'b');xlabel('Year'); ylabel('Carbonate loss (cm)')

% at 1 kg CACO3 m2 year is 0.75 mm

a= .075; bS= .1; cD= .45;

[t A] = ode45(fname, timespan, y0); plot(t, A,'r')

% at 10 kg CACO3 m2 year is 7 mm

a= .7; bS= .1; cD= .45; [t A] = ode45(fname, timespan, y0);

plot(t, A,'g')

% For imperforate skeletons change cD to 0.1 above
